# A spatially interpretable machine learning framework for urban waterlogging risk mapping in Beijing

**DOI:** 10.7717/peerj.20977

**Published:** 2026-03-19

**Authors:** Yi Tang

**Affiliations:** 1 School of Emergency Technology and Management, Institute of Disaster Prevention, Sanhe, China; 2 Hebei Key Laboratory of Resource and Environmental Disaster Mechanism and Risk Monitoring, Sanhe, China; 3 Department of Statistics and Data Science, Tsinghua University, Beijing, China

**Keywords:** Emergency management, Flood, Flood prediction, GEE, Spatial machine learning, Spatial risk mapping

## Abstract

Urban waterlogging is an escalating challenge under rapid urbanization and climate change, yet accurate spatial prediction remains hindered by nonlinear drivers and spatial heterogeneity. This study proposes a spatially interpretable machine learning framework by integrating remote sensing and geospatial data with hybrid modeling. Using recorded waterlogging locations in Beijing, we constructed a balanced dataset with topographic, hydrological, land cover, and proximity-based predictors. Four machine learning algorithms—Random Forest (RF), Support Vector Machine (SVM), K-Nearest Neighbor (KNN), and eXtreme Gradient Boosting (XGBoost)—were evaluated, with XGBoost achieving the best classification performance (area under the curve (AUC) = 0.913 ± 0.055). To enhance spatial interpretability, two hybrid strategies were further developed: (1) XGBoost_MGWR, in which XGBoost serves as the primary predictor and MGWR corrects its spatially structured residuals, thereby improving spatial explanatory power; and (2) MGWR_XGBoost, where MGWR first models spatially varying effects and XGBoost subsequently fits the residuals to refine predictive performance. Results from spatially blocked five-fold cross-validation show that MGWR_XGBoost provides the best probabilistic accuracy (Brier = 0.289 ± 0.039) and the highest area under the precision recall (PR-AUC) (0.576), with substantially higher specificity (0.734) and a spatially stable local *R*^2^ pattern; therefore, it was selected for final risk mapping. The proposed framework enables high-resolution, spatially explicit risk mapping and offers practical support for drainage planning, green infrastructure prioritization, and adaptive flood governance. Beyond Beijing, this approach shows strong potential for improving resilience in other data-scarce urban environments facing intensifying flood risks.

## Introduction

Global climate change has intensified the hydrological cycle, producing increasingly frequent and severe precipitation extremes (Bengtsson, 2010; Wasko et al., 2021; Faranda et al., 2024). These short-duration, high-intensity rainfall events pose escalating threats to ecosystems, infrastructure, and human safety, with the impacts being particularly severe in densely populated urban areas. Among hydrometeorological hazards, urban waterlogging and pluvial inundation have emerged as some of the most recurrent and disruptive phenomena (Palla et al., 2018; Singh, Nielsen & Greatrex, 2023). In modern cities, the expansion of impervious surfaces, inadequate drainage networks, and rapid urbanization converge to amplify runoff generation (Yu et al., 2018). As a result, even moderate rainfall can overwhelm urban drainage systems, while extreme events frequently trigger widespread surface inundation, leading to transport disruptions, infrastructure damage, and public safety hazards (Qin, 2020). These trends highlight the urgent need for predictive, spatially adaptive tools to inform flood-resilient urban planning and risk-sensitive disaster governance.

Traditionally, hydrological simulation tools such as the Storm Water Management Model (SWMM) and empirical approaches like multiple linear regression have been widely applied for urban flood prediction (Ahmad et al., 2024). These methods provide useful insights into drainage processes, but their reliance on fixed assumptions, extensive calibration, and site-specific parameters limits their scalability and applicability in complex, data-limited urban environments.

To overcome these limitations, spatial regression models such as Geographically Weighted Regression (GWR) and Multiscale Geographically Weighted Regression (MGWR) have been increasingly employed in urban inundation studies (Ali et al., 2024; Mandal & Goswami, 2025). By allowing regression coefficients to vary across space, these models capture geographic heterogeneity in flood-driving factors, and MGWR further enhances flexibility by enabling each variable to operate at its own spatial scale. Nonetheless, as linear frameworks, GWR and MGWR struggle to account for nonlinear interactions among predictors, and their predictive accuracy often falls behind that of more advanced machine learning approaches.

Machine learning (ML) techniques such as Random Forest (RF), Support Vector Machine (SVM), and eXtreme Gradient Boosting (XGBoost) have demonstrated strong performance in identifying inundation-prone areas using high-dimensional environmental data (Li et al., 2025). Their predictive power, however, typically comes at the expense of interpretability, as most ML models treat space implicitly and assume stationarity, limiting their ability to explain localized variations in flood risk.

Recent efforts have attempted to integrate ML with spatial regression to combine predictive accuracy with spatial interpretability (Yang et al., 2022; Shen et al., 2024). Yet, most studies adopt a single hybrid pathway—typically using ML outputs as inputs for GWR or MGWR—while the comparative performance of alternative hybrid structures remains largely unexplored. Moreover, the trade-off between global accuracy and local explanatory power has seldom been explicitly addressed, particularly for spatially sparse urban flood datasets.

This study seeks to fill these gaps by systematically comparing two hybrid strategies: (1) XGBoost_MGWR, in which XGBoost serves as the primary predictor and MGWR models the spatially structured residuals to provide spatial interpretation, and (2) MGWR_XGBoost, where MGWR residuals are modeled by XGBoost to enhance prediction accuracy. We evaluate both strategies in terms of predictive performance and spatial explanatory power, aiming to provide a high-accuracy and spatially interpretable framework for urban flood risk mapping in data-scarce environments.

## Material and Method

### Study area

Beijing, the capital of the People’s Republic of China, is located in northern China and serves as the nation’s political, cultural, and economic center. Over the past few decades, the city has experienced rapid urbanization, which has led to significant land use transformation and a marked increase in impervious surface coverage. These changes, coupled with aging and unevenly distributed drainage infrastructure, have substantially elevated the risk of urban waterlogging during intense rainfall events.

Geographically, Beijing lies in a semi-arid monsoonal climate zone characterized by hot, humid summers and cold, dry winters. Most of the annual precipitation occurs during the summer months, often in the form of short-duration, high-intensity rainfall. The city is situated in a basin flanked by mountains to the west, north, and northeast, which impedes natural runoff and promotes water accumulation in low-lying urban areas.

**Figure 1:**
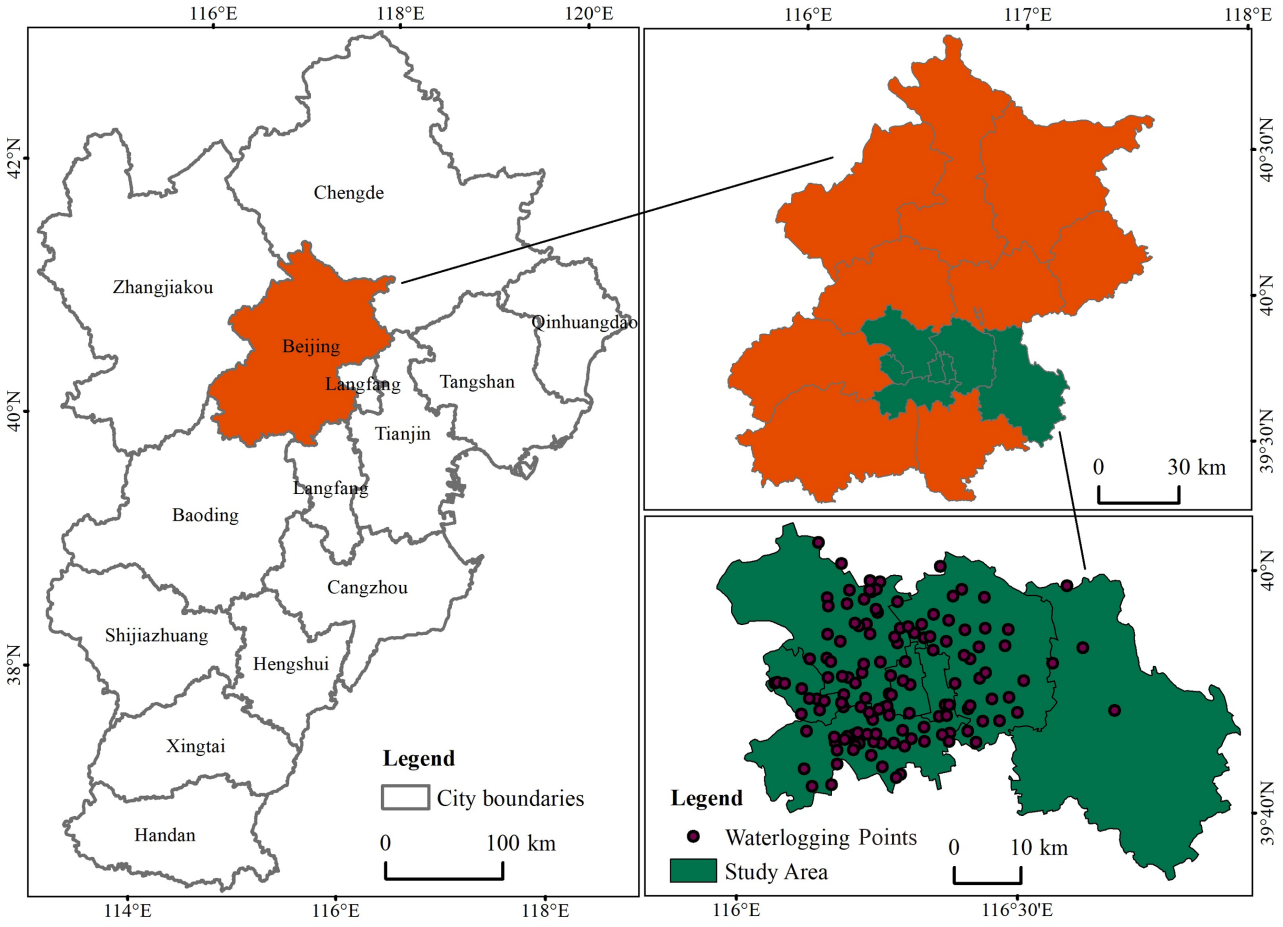
Location of the study area.

This study focuses on the urban core of Beijing, encompassing eight densely developed districts: Dongcheng, Xicheng, Chaoyang, Haidian, Fengtai, Shijingshan, Tongzhou, and Changping (Fig. 1). These districts represent the city’s most urbanized zones, characterized by high population density, complex road networks, and significant exposure to flood hazards. Due to their topographic constraints and infrastructural vulnerabilities, these areas have historically exhibited concentrated occurrences of pluvial flooding, making them a critical focus for spatially refined flood risk assessment and mitigation.

## Methodological Framework

**Figure 2:**
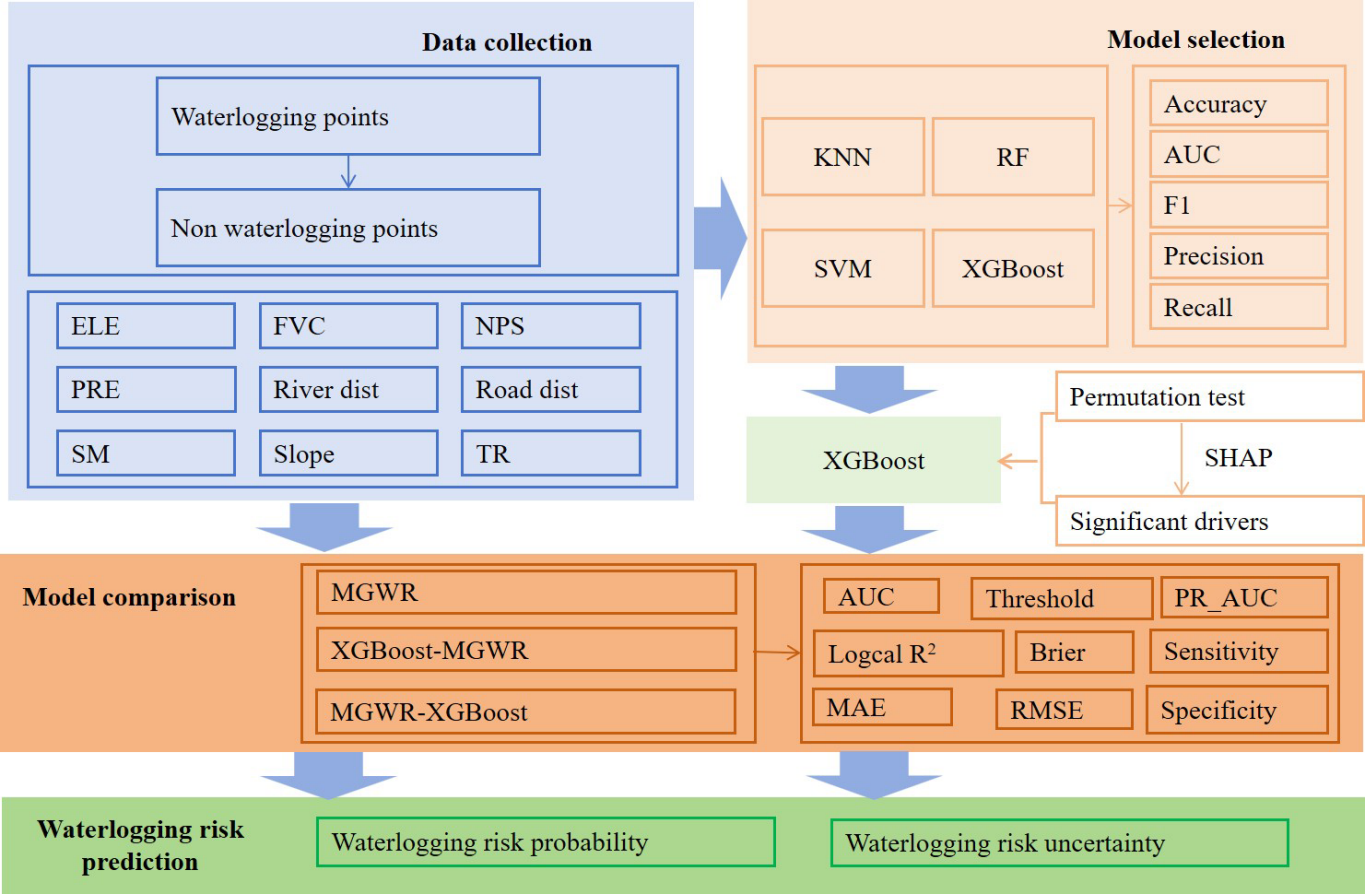
Comparison of three modeling strategies for urban waterlogging prediction.

This study employed a four-stage methodological framework to predict urban waterlogging risk (Fig. 2). First, waterlogging and non-waterlogging points were identified and labeled to form a binary classification dataset. A set of environmental predictors was extracted as input variables. Second, four machine learning models were evaluated using metrics. Feature importance was then assessed using permutation tests and SHAP values to identify significant drivers. Based on the cross-validated performance, XGBoost was selected as the representative machine-learning algorithm for constructing the subsequent hybrid models. Third, to improve spatial interpretability, a hybrid modeling strategy was adopted. We compared three approaches: MGWR alone, XGBoost followed by MGWR (XGBoost_MGWR), and MGWR followed by XGBoost residual correction (MGWR_XGBoost). These models were evaluated using statistical and spatial metrics. Finally, the optimal model was used to produce a high-resolution waterlogging risk map, providing scientific support for targeted flood mitigation and urban resilience planning.

### Data collection

The primary dataset used in this study is the spatial distribution of historical urban waterlogging points, obtained from the Urban Waterlogging Map (http://www.bjdx.gov.cn). A total of 139 typical waterlogging locations were identified based on past extreme rainfall events and municipal drainage records. These points serve as positive samples (label = 1) in the machine learning classification model. Their spatial distribution covers eight high-risk urban districts—Dongcheng, Xicheng, Chaoyang, Haidian, Fengtai, Shijingshan, Tongzhou, and Changping—representing the core urbanized areas of Beijing. To generate corresponding negative samples (label = 0), non-waterlogging points were created using spatial buffering and distance-based exclusion techniques to avoid spatial autocorrelation and ensure data balance.

To construct predictive features, a suite of environmental variables was derived from the Google Earth Engine (GEE) platform. Elevation (ELE) data were obtained from the Digital Elevation Model and were subsequently used to compute Slope and Terrain Ruggedness (TR), both of which characterize terrain complexity and surface drainage potential (Table 1).

**Table 1:** Data sources.

Indicators	Abbreviation	Sources	Resolution	Resampling
Elevation	ELE	USGS/SRTMGL1_003	30 m	None (template)
Slope	Slope	USGS/SRTMGL1_003	30 m	None
Terrain Ruggedness	TR	USGS/SRTMGL1_003	30 m	None
Precipitation	PRE	UCSB-CHG/CHIRPS/DAILY	0.05°	Bilinear
Soil Moisture	SM	NASA/SMAP/SPL4SMGP/007	9 km	Bilinear
Fractional Vegetation Cover	FVC	MODIS/006/MOD13Q1	250 m	Bilinear
Non-Permeable Surface	NPS	http://zenodo.org/records/5220816	30 m	none
Distance to River	River_dist	http://www.openstreetmap.org	Vector	Rasterization
Distance to Road	Road_dist	http://www.openstreetmap.org	Vector	Rasterization

Precipitation (PRE) was derived from the CHIRPS (Climate Hazards Group InfraRed Precipitation with Station data) daily rainfall product. Given that the flooded points were identified based on events over the past decade, we computed the average annual precipitation over the 2011–2021 period to represent long-term climatic rainfall patterns. This 11-year mean serves as a robust baseline for hydrological modeling and avoids biases introduced by short-term anomalies.

Soil moisture (SM) was derived from the SMAP Level-4 (L4) soil moisture product, and we computed the multi-year mean over 2015–2021, as SMAP observations are only available from 2015 onward (Reichle et al., 2025). It represents root-zone water availability that influences infiltration and runoff dynamics. Fractional Vegetation Cover (FVC) was derived from NDVI using a dynamic thresholding method based on the 5th and 95th percentiles, and similarly averaged over 2011–2021. This metric captures the regulatory role of vegetation in surface water retention and evapotranspiration. Non-Permeable Surface (NPS) was extracted from the GISD30 dataset (Global 30-m Impervious Surface Dynamic Dataset, 1985–2020), which quantifies impervious surface dynamics using time-series Landsat imagery on the Google Earth Engine platform (Liu et al., 2021). We selected the latest available year (2020) to represent urbanization-induced impervious surfaces. All environmental raster layers were first reprojected from their native geographic coordinates (WGS84) to a common projected coordinate system (WGS84/UTM Zone 50N, EPSG:32650) and clipped to the study area. To characterize drainage and flow pathways, two proximity variables were included: distance to the nearest river (River_dist) and distance to the nearest road (Road_dist). These were computed from vector layers obtained from OpenStreetMap (accessed: 23 Apr 2025; ©OpenStreetMap contributors), using Euclidean distance calculations. River proximity reflects hydrological connectivity and drainage potential, while road proximity may indicate areas where drainage is obstructed or surface water is concentrated.

To ensure the representativeness and balance of the dataset, we constructed a binary classification sample by pairing actual waterlogging points with an equal number of non- waterlogging control points. Specifically, non- waterlogging points were randomly generated within the study area boundaries, with the constraint that they had to be at least three km away from any flooded point in order to reduce local spatial dependence between cases and controls. To assess the sensitivity of this negative-sampling scheme, we repeated the sampling and 10-fold cross-validation using minimum separation distances of one km and two km; the corresponding area under the curve (AUC), root mean square deviation (RMSD), mean absolute error (MAE) and Brier scores are reported in Table S1. Across the three distance thresholds, model performance remained broadly consistent and improved slightly as the buffer increased, indicating that the proposed modelling framework is robust to the choice of minimum case–control separation distance. For each selected non-waterlogging location, we extracted the same set of environmental variables used for flooded points. This process yielded a balanced dataset consisting of 139 flooded and 139 non-flooded points.

### Machine learning models

Based on the constructed labeled dataset, we evaluated and compared four widely used supervised machine learning algorithms: RF, K-Nearest Neighbors (KNN), XGBoost, and SVM. Each algorithm was selected based on its particular strengths in modeling environmental systems and handling structured data in binary classification tasks.

RF was implemented using the rf engine in the *caret* package, which wraps the standard random Forest algorithm (Kuhn, 2008). All trees used the default splitting rule based on the Gini impurity. The number of trees was kept at the package default (500 trees), while the number of variables randomly sampled at each split (*mtry*) was tuned by *caret* over five candidate values automatically generated from the predictor set. The optimal *mtry* value was selected by maximizing the cross-validated AUC. Variable importance was extracted from the fitted RF model using the mean decrease in the Gini index, and was later used as a descriptive measure of each predictor’s contribution.

KNN classification was implemented with the knn method in caret, using the Euclidean distance metric. Before model fitting, all predictors were centered and scaled to zero mean and unit variance to avoid distortions in distance computation due to differing units. The number of neighbors (k) was tuned by caret over an automatically generated grid of integer values (tuneLength = 5), and the value yielding the highest cross-validated AUC was retained as the final model.

XGBoost was implemented with the xgbTree method in caret package. A small, structured hyperparameter grid was specified a priori: the number of boosting rounds was fixed at 100 (nrounds = 100); the maximum tree depth (max_depth) was allowed to vary between 2, 3 and 4 to control model complexity; the learning rate (eta) took values 0.05 and 0.10; the minimum child weight was set to 5 or 10; and a tree-splitting penalty (gamma) of 0.1 or 0.5 was imposed. The column subsampling rate (colsample_bytree) and row subsampling rate (subsample) were both set to 0.8. All combinations in this grid were evaluated under 10-fold cross-validation, and the configuration achieving the highest AUC was chosen. The underlying XGBoost implementation used a binary logistic objective (“binary:logistic”) to output class probabilities.

SVM was trained using the SVM Radial method in caret, which corresponds to a C-support vector classifier with a radial basis function (RBF) kernel. The two key hyperparameters—the cost parameter (C) and the kernel width (γ)—were tuned jointly over a data-driven grid of five combinations (tune length = 5) defined on a logarithmic scale by caret. Hyperparameter selection was again based on maximizing the 10-fold cross-validated AUC, and the final SVM model was refitted on the full dataset using the optimal (C, γ) pair.

Because the sample size is relatively limited and the dataset was artificially balanced, we did not reserve a separate hold-out test set. Instead, all machine-learning models were evaluated using stratified 10-fold cross-validation on the full dataset. In each fold, 90% of the data were used for model fitting and hyperparameter tuning and the remaining 10% for validation, while preserving the proportion of flooded and non-flooded samples. Predictive performance across folds was summarized as mean ± standard deviation for the area under the receiver operating characteristic (ROC) curve (AUC), Accuracy, Precision, Recall, and F1-score of the pure ML classifiers (KNN, RF, SVM, and XGBoost).

To identify the most influential predictors and improve model interpretability, we conducted permutation importance analysis and SHAP (SHapley Additive exPlanations) analysis on the trained XGBoost model. For permutation importance, we used the cross-validated XGBoost model fitted on the full dataset and took the AUC as the test statistic. For each candidate predictor, we generated *n* = 100 permutations by randomly shuffling its values while keeping all other variables and the fitted model fixed, recomputed the AUC for each permuted dataset, and calculated the difference ΔAUC = AUC_original_− AUC_permuted_. The Monte Carlo *p*-value for a given predictor was estimated as the proportion of permutations for which ΔAUC ≤ 0, *i.e.,* where the permuted AUC was at least as large as the original AUC. A conventional type-I error level of 0.05 was adopted, and predictors with *p* < 0.05 were treated as statistically important. Based on these results, a reduced XGBoost model was retrained using only the statistically significant predictors to eliminate redundancy and potential noise. SHAP analysis was then applied to the reduced model, using the original dataset as background, to quantify the marginal contribution of each predictor to individual waterlogging-risk predictions and to characterize how these contributions vary across observations.

### MGWR

To capture spatial heterogeneity in the relationship between environmental covariates and flood risk, we employed the MGWR model, which allows each predictor to operate at its own optimal spatial bandwidth. The model was implemented in R using the GWmodel package (Gollini et al., 2015) as a local linear regression (Gaussian/identity specification) with the binary response coded as 0/1. MGWR yields a location-specific linear predictor *η*_i_, which we subsequently mapped to the [0,1] interval using a logistic transformation to obtain a probabilistic flood susceptibility score:

(1) \begin{eqnarray*}{\eta }_{i}={\beta }_{0} \left( {s}_{i} \right) +\sum _{j=1}^{p}{\beta }_{j} \left( {s}_{i} \right) \cdot {x}_{ij}\end{eqnarray*}

(2) \begin{eqnarray*}{p}_{i}^{MGWR}= \frac{1}{1+exp \left( -{\eta }_{i}^{MGWR} \right) } \end{eqnarray*}

where *s*_i_ = (*u*_i_,*v*_i_) denotes the spatial coordinates of location *i*; β_j_(*s*_i_) is the local coefficient of the *j*-th covariate at location *i*, estimated using its own optimal bandwidth bw_j_; and *x*_ij_ is the standardized value of the *j*-th environmental variable at location *i*.

An adaptive bandwidth strategy was used to accommodate spatially uneven data density. Four kernel functions—bisquare, Gaussian, exponential and tricube—were tested (diagnostics reported in Table S2). The bisquare kernel (optimal bandwidth = 67) provided the lowest AIC and RSS and the highest *R*^2^ and adjusted *R*^2^, and was therefore selected for the final MGWR model.

Prior to model fitting, we conducted multicollinearity diagnostics among the candidate environmental variables using the Variance Inflation Factor (VIF). Variables with VIF values exceeding the commonly accepted threshold of 10 were considered highly collinear and were removed to prevent instability in coefficient estimation and reduce model redundancy. In addition, we performed local multicollinearity diagnostics in GWmodel to compute the local condition number at each location and identify areas with potential severe local multicollinearity (*e.g.*, local condition number > 30). Finally, to verify that MGWR adequately accounted for spatial dependence, we calculated Moran’s I for the MGWR residuals using an 8–nearest neighbours (k–NN) spatial weights matrix and the moran.test function in the spdep package, and assessed the significance of any remaining spatial autocorrelation.

### XGBoost_MGWR

To improve predictive performance while retaining spatial interpretability, we developed an XGBoost–MGWR hybrid. XGBoost serves as the primary predictor by capturing global nonlinear relationships, and MGWR models the remaining spatially structured lack-of-fit on the logit/score (margin) scale. Let *yi* ∈ {0, 1} denote the observed flood occurrence at location *i*, *s*_i_ = (*u*_i_,v_i_) denote its spatial coordinates, and *x*_ij_ denote the standardized value of covariate *j* (*j* = 1, …, *p*) at location *i*.

Let *p*_i_^XGB^ be the XGBoost predicted probability at location i, and Z_i_^XGB^ = logit(*p*_i_^XGB^) the corresponding score (log-odds). To ensure scale consistency, we define a working residual on the score scale (first-order Newton/IRLS approximation to the logistic loss):

(3) \begin{eqnarray*}\Delta {\eta }_{i}^{XGB}= \frac{{y}_{i}-{p}_{i}^{XGB}}{{p}_{i}^{XGB}(1-{p}_{i}^{XGB})} \end{eqnarray*}

MGWR is then fitted to $\Delta {\eta }_{i}^{XGB}$ to learn a spatial correction term:

(4) \begin{eqnarray*}{\widehat{\Delta \eta }}_{i}^{MGWR}={\alpha }_{0} \left( {s}_{i} \right) +\sum _{j=1}^{p}{\alpha }_{j} \left( {s}_{i} \right) \cdot {x}_{ij}\end{eqnarray*}

The final combined score and probability are:

(5) \begin{eqnarray*}{S}_{i}^{XGB\mathrm{_}MGWR}& ={Z}_{i}^{XGB}+{\widehat{\Delta \eta }}_{i}^{MGWR}\end{eqnarray*}

(6) \begin{eqnarray*}{p}_{i}^{XGB\mathrm{_}MGWR}& = \frac{1}{1+exp \left( -{S}_{i}^{XGB\mathrm{_}MGWR} \right) } \end{eqnarray*}

In implementation, probabilities were clipped to (*ɛ*,1−*ɛ*) before applying logit to avoid numerical instability.

### MGWR_XGBoost

We implemented an alternative hybrid framework, referred to as the MGWR_XGBoost model, in which MGWR provides the primary spatially varying linear predictor and XGBoost supplies an additive correction on the score scale. MGWR was first yields:

(7) \begin{eqnarray*}{\eta }_{i}^{MGWR}& ={\beta }_{0} \left( {s}_{i} \right) +\sum _{j=1}^{p}{\beta }_{j} \left( {s}_{i} \right) \cdot {x}_{ij}\end{eqnarray*}

(8) \begin{eqnarray*}{p}_{i}^{MGWR}& = \frac{1}{1+exp \left( -{\eta }_{i}^{MGWR} \right) } \end{eqnarray*}

We then define a working residual on the score scale:

(9) \begin{eqnarray*}\Delta {\eta }_{i}^{MGWR}= \frac{{y}_{i}-{p}_{i}^{MGWR}}{{p}_{i}^{MGWR}(1-{p}_{i}^{MGWR})} \end{eqnarray*}

XGBoost is trained as a regression model to learn ${\widehat{\Delta \eta }}_{i}^{XGB}=\mathrm{fXGB}(\mathrm{xi})$. *f*_XGB_ (*x*_i_) denotes the additive correction learned by XGBoost as a function of covariates *x*_i_. The final combined score and probability are:

(10) \begin{eqnarray*}{S}_{i}^{MGWR\mathrm{_}XGB}& ={\eta }_{i}^{MGWR}+{\widehat{\Delta \eta }}_{i}^{XGB}\end{eqnarray*}

(11) \begin{eqnarray*}{p}_{i}^{MGWR\mathrm{_}XGB}& = \frac{1}{1+exp \left( -{S}_{i}^{MGWR\mathrm{_}XGB} \right) } \end{eqnarray*}

where ${p}_{i}^{MGWR\mathrm{_}XGB}$ denotes the predicted probability of waterlogging at location *i*, ${\eta }_{i}^{MGWR}$ is the MGWR linear predictor evaluated at s_i_.

### Model performance evaluation

For MGWR and both hybrid pipelines (XGBoost_MGWR and MGWR_XGBoost), waterlogging prediction was formulated as a probabilistic binary classification problem. Model performance was evaluated using a spatially blocked five-fold cross-validation scheme to reduce spatial leakage. Specifically, all observations were partitioned into five (*K* = 5) spatial folds using k-means clustering on projected point coordinates, and each spatial cluster was treated as one held-out block. For each fold, models were trained on the remaining four spatial blocks and evaluated on the held-out block, ensuring that all training steps—including residual learning in the hybrid pipelines—used training data only.

Specifically, within each spatial fold, the primary learner (MGWR or XGBoost) was fitted using the training blocks only; residuals were computed on the training data only; and the residual-correction learner (MGWR or XGBoost) was then trained using these training residuals only. The held-out block was predicted by combining the primary prediction with the residual correction, yielding out-of-fold probabilities for that block. We did not implement a separate nested cross-validation layer; instead, MGWR bandwidth selection was carried out within each training fold, and XGBoost was refitted within each fold using a fixed parameter setting. Finally, we concatenated the held-out predictions across folds so that each observation has exactly one out-of-fold prediction used for performance evaluation.

Within each fold, we quantified discrimination and probabilistic accuracy using ROC-AUC, the Brier score (mean squared error of predicted probabilities), RMSE (square root of the Brier score), and MAE between predicted probabilities and the 0/1 flood labels. Fold-wise metrics were summarized as mean ± standard deviation to characterize average performance and its variability across spatial partitions. To obtain a single cross-validated prediction for each observation, we concatenated the out-of-fold predicted probabilities from all held-out blocks. Using this pooled set of out-of-fold predictions, we additionally computed the area under the precision–recall curve (PR_AUC) as a complementary discrimination metric emphasizing the flooded class, and identified a Youden-optimal decision threshold on the ROC curve, reporting the corresponding sensitivity and specificity. Given the balanced case–control sampling design, these threshold-based metrics provide an interpretable trade-off between detecting flooded locations (sensitivity) and limiting false alarms (specificity). Finally, for MGWR we mapped the local *R*^2^ statistics to examine how explanatory power varies spatially and to support spatially explicit interpretation of covariate effects.

### Waterlogging risk map

Based on spatial five-fold cross-validation and local *R*^2^ diagnostics, we selected MGWR_XGBoost to generate the final risk map, as it provides more reliable probability estimates while maintaining a spatially consistent fit.

For wall-to-wall mapping, all predictor layers were first harmonized to a common 30 m grid in UTM Zone 50 N using elevation as the template, resampled as needed, and masked to the study boundary. Grid-cell covariates were then standardized using the mean and standard deviation from the modelling dataset to ensure identical scaling to model fitting.

Spatial prediction under the MGWR_XGBoost framework was implemented in two components. The MGWR provided a baseline score surface by interpolating the fitted MGWR values from observation locations to all grid cells using a thin-plate spline. The XGBoost residual model then produced an additive correction score for each grid cell from the standardized covariates. These two components were combined and transformed through the logistic link to obtain cell-wise waterlogging probabilities.

The continuous probability surface was subsequently discretized into three waterlogging risk levels, *i.e.,* low risk (*p* < 0.50), moderate risk (0.50 ≤*p* < 0.70), and high risk (*p* ≥ 0.70). The lower cut-off of 0.50 is close to the optimal Youden threshold obtained from spatial cross-validation, while the 0.70 cut-off highlights high-confidence hotspots of waterlogging risk.

To quantify prediction uncertainty, we further derived an uncertainty index for each grid cell as *u* = *p* (1−*p*). This measure attains its maximum around *p* = 0.5, indicating uncertain predictions, and approaches zero when the model is highly confident (probabilities close to 0 or 1). For generating continuous prediction maps, we used the elevation raster as a template and resampled all continuous covariates to the same 30 m UTM grid using bilinear interpolation, while proximity variables were computed from vector layers *via* Euclidean distance rasters, before stacking them into a multi-layer raster for spatial prediction.

## Results

### Model selection

The performance of four machine-learning models was shown in Table 2. Overall, the tree-based ensemble models clearly outperformed KNN and SVM. XGBoost achieved the highest accuracy (0.820 ± 0.065) and the highest AUC (0.913 ± 0.055), indicating the best overall discrimination between flooded and non-flooded locations. Its F1-score (0.819 ± 0.071) and Precision (0.820 ± 0.065) were also the highest among all models, suggesting that XGBoost provided the most balanced trade-off between correctly identifying flooded cells and avoiding false alarms.

**Table 2:** ML models performance comparison.

Model	Accuracy	AUC	F1-score	Precision	Recall
KNN	0.730 ± 0.06	0.806 ± 0.045	0.752 ± 0.046	0.705 ± 0.074	0.813 ± 0.061
RF	0.813 ± 0.068	0.894 ± 0.062	0.816 ± 0.073	0.805 ± 0.088	0.841 ± 0.123
SVM	0.763 ± 0.078	0.824 ± 0.074	0.761 ± 0.085	0.764 ± 0.086	0.769 ± 0.131
XGBoost	0.820 ± 0.065	0.913 ± 0.055	0.819 ± 0.071	0.820 ± 0.065	0.826 ± 0.115

Random Forest showed very competitive performance, with accuracy of 0.813 ± 0.068 and AUC of 0.894 ± 0.062. Its Recall (0.841 ± 0.123) was slightly higher than that of XGBoost (0.826 ± 0.115), implying a marginally stronger tendency to capture flooded locations at the cost of a small reduction in precision. In contrast, KNN and SVM exhibited lower overall performance, with AUC values of 0.806 ± 0.045 and 0.824 ± 0.074 and F1-scores of 0.752 ± 0.046 and 0.761 ± 0.085, respectively. These results indicate weaker discrimination and less favorable precision–recall trade-offs compared with the ensemble-based models.

### Relative importance of variables

**Figure 3:**
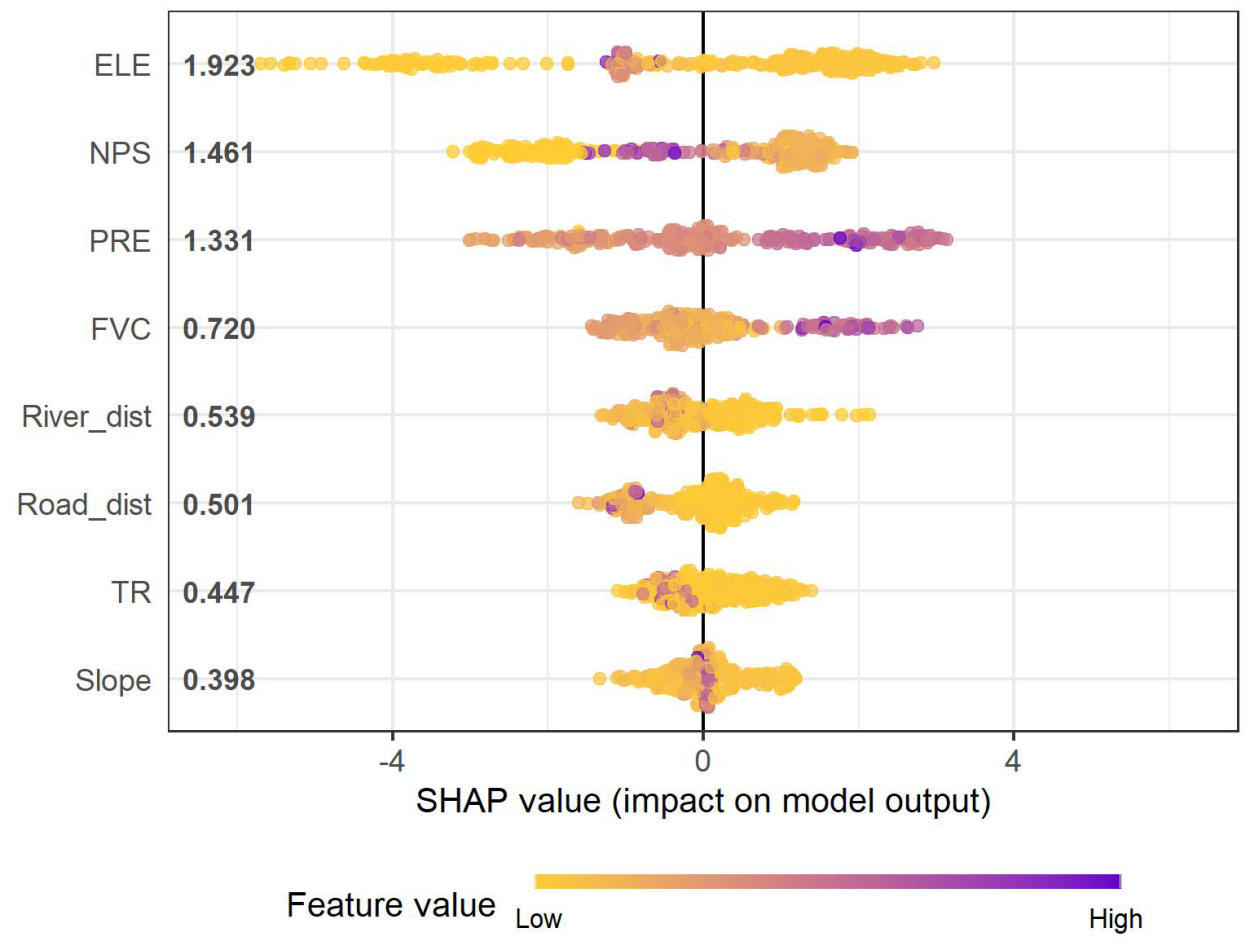
SHAP summary plot of predictor contributions.

Permutation testing revealed that only eight variables were statistically significant (*p* < 0.05) in contributing to the prediction of flood occurrence: ELE, NPS, PRE, FVC, River_dist, Road_dist, TR and Slope. The SHAP summary plot visualizes the distribution of signed contributions across samples, while we use mean absolute SHAP values to rank global predictor importance (Fig. 3).

 Among all variables, ELE had the highest SHAP importance (1.923). NPS and PRE ranked second and third with SHAP values of 1.461 and 1.331, respectively. FVC and River_dist and also showed substantial importance, with SHAP values of 0.720 and 0.539. Road_dist, TR and Slope exhibited the lowest but still non-negligible SHAP importance.

**Figure 4:**
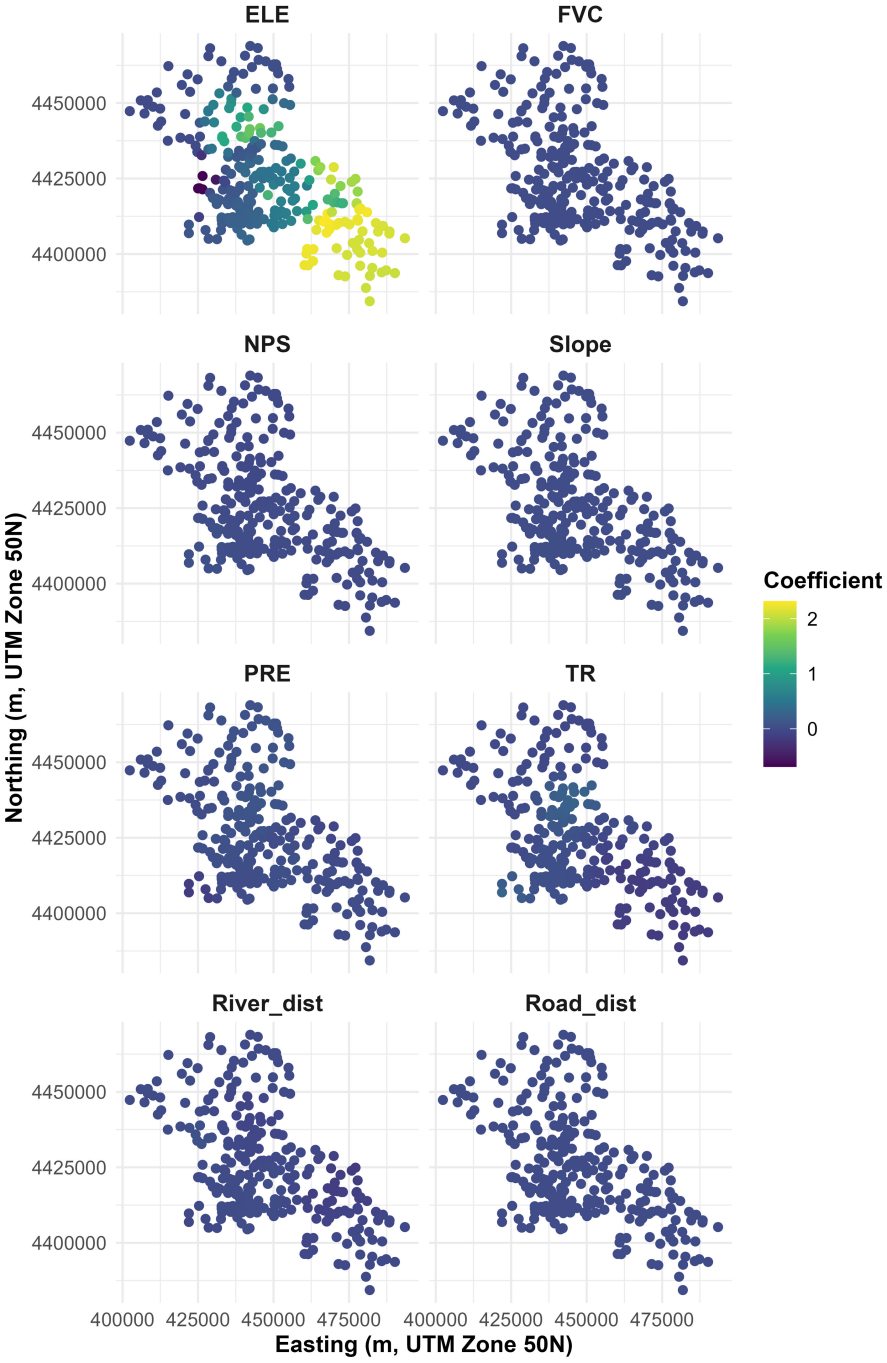
The spatial distribution of local coefficients of environmental predictors based on the MGWR model.

### Spatial heterogeneity of predictor effects (MGWR Model)

Eight predictors were retained in the MGWR analysis, with SM excluded due to strong multicollinearity (VIF > 10). The local coefficient surfaces indicate that elevation (ELE) exhibits the most pronounced spatial heterogeneity and the largest effect sizes (Fig. 4). Its coefficients are generally positive but increase markedly toward the southeastern sector, while remaining comparatively weak (and locally near-zero to slightly negative) in other parts of the study area. In contrast, FVC, NPS, Slope, PRE, and Road_dist display coefficients that are tightly concentrated around zero and are spatially smooth across most locations, suggesting limited spatial differentiation in their marginal effects. Besides, TR shows modest spatial gradients with coefficients shifting from weakly positive in parts of the central area to weakly negative toward the periphery (Fig. 4).

**Figure 5:**
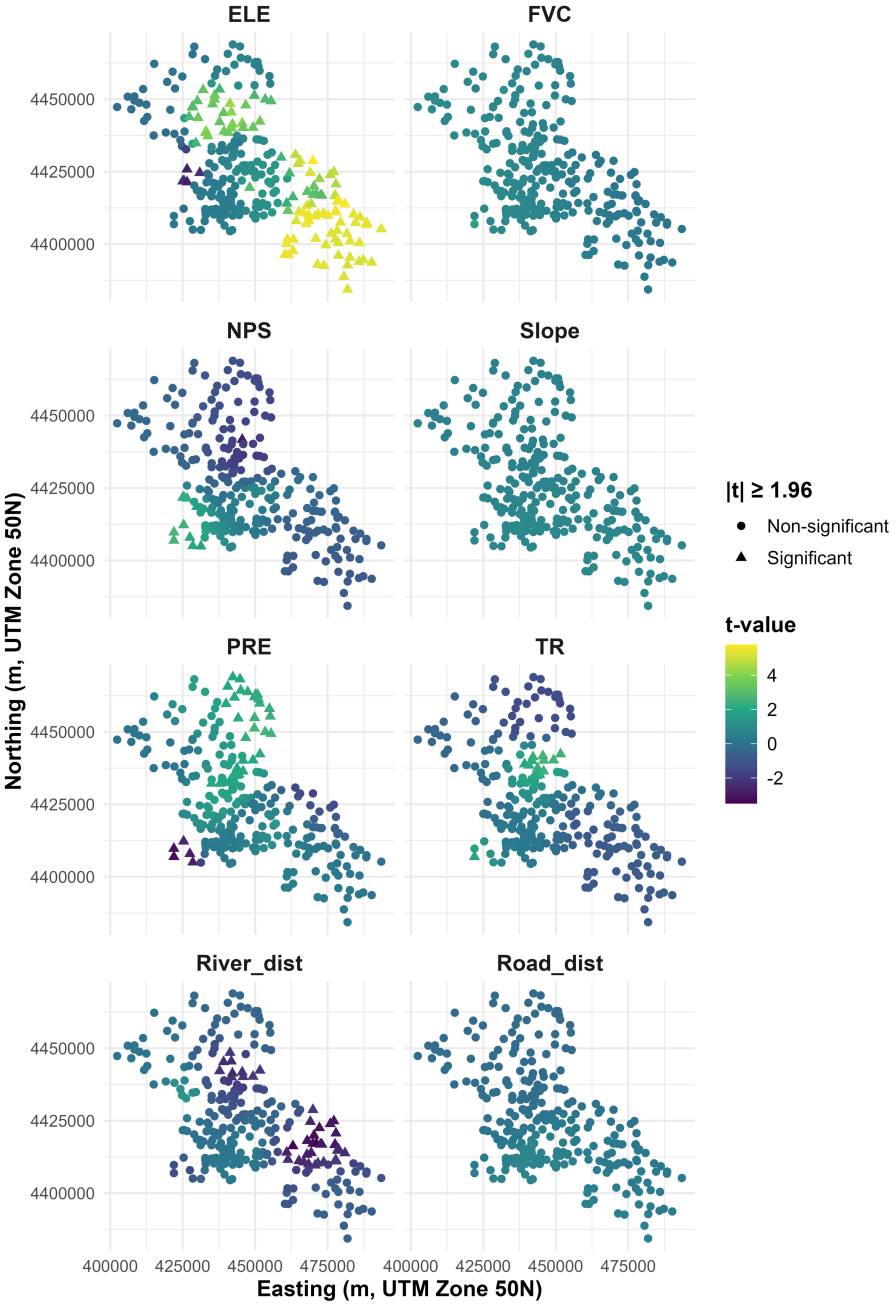
Spatial distribution of t-values for predictors based on MGWR.

The *t*-value maps indicate where the spatially varying MGWR coefficients are statistically distinguishable from zero (|*t*|≥ 1.96; Fig. 5). ELE shows the strongest and most spatially coherent pattern, with predominantly significant positive coefficients in the north-central high-elevation cluster and the southeastern corridor, plus a small pocket of negative significance in the central–northern area. In contrast, FVC, Slope, and Road_dist are largely non-significant, with *t*-values close to zero across most sites. NPS shows localized sign changes, characterized by a small cluster of significant positive coefficients in the central–southwestern area and only a few isolated significant negative locations. PRE exhibits a mixed pattern, with significant positive coefficients in parts of the north-central region and significant negative coefficients at the southwestern edge. TR presents a localized cluster of significant positive coefficients in the central area, while most other locations remain weak or non-significant. River_dist shows a distinct localized negative association, with significant negative *t*-values concentrated in the central–northern and southeastern parts of the study area (Fig. 5).

**Figure 6:**
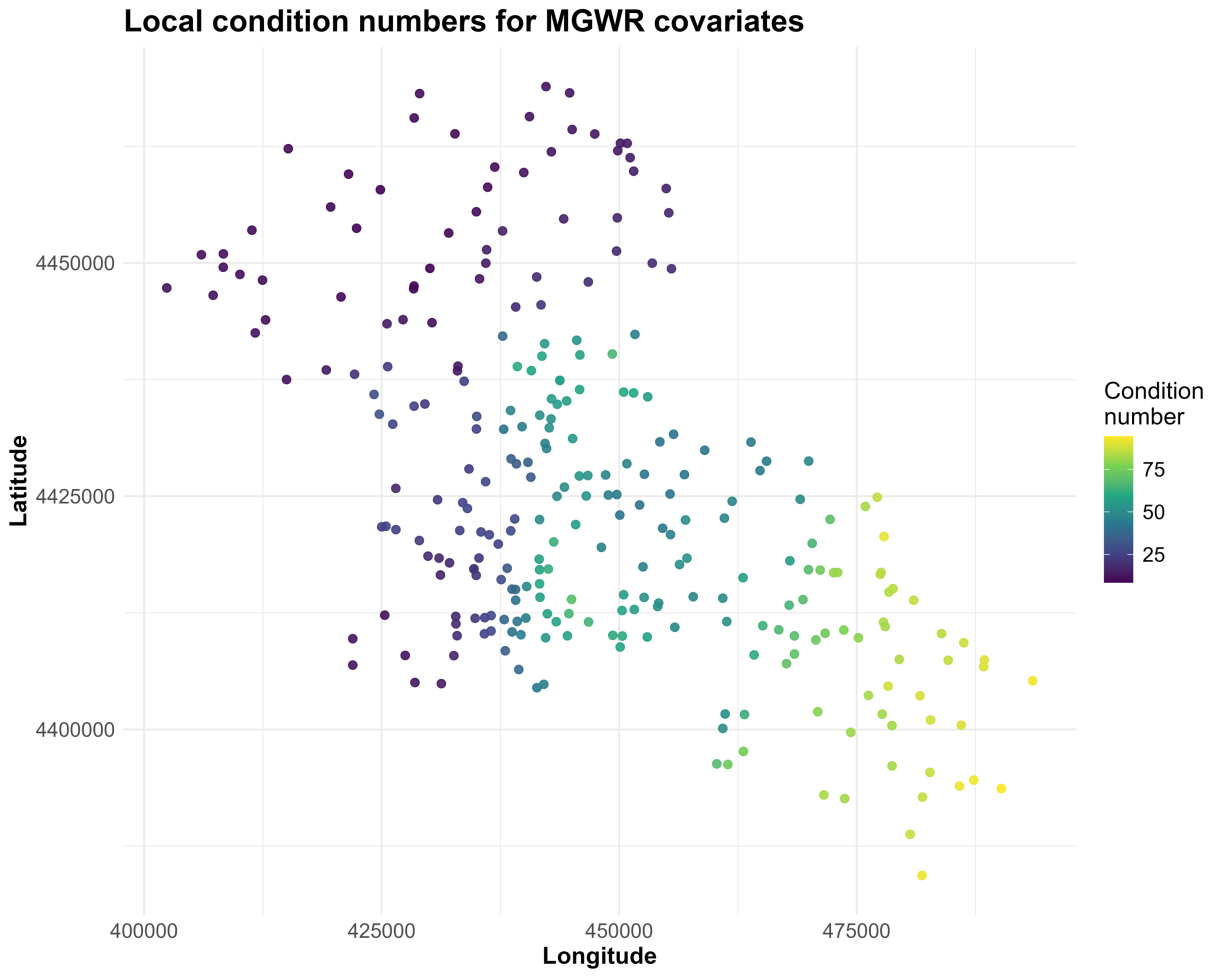
Spatial distribution of local condition numbers for the MGWR covariates, with warmer colours indicating areas of stronger local multicollinearity.

Local condition numbers for the MGWR covariates showed clear spatial variation (Fig. 6). Values were generally low in the north-western part of the study area (predominantly < 30), increased to moderate levels across the central corridor (approximately 30–60), and reached their highest levels in the south-eastern sector, where many sites exceeded 60 and some approached ∼80–90. This pattern indicates that local multicollinearity is spatially concentrated in the south-eastern/downstream zone, whereas most other locations fall within ranges commonly considered acceptable for MGWR inference. Accordingly, coefficient estimates in the south-eastern cluster should be interpreted with greater caution due to the heightened risk of unstable local parameter estimates driven by stronger predictor collinearity.

Global Moran’s I computed on the MGWR residuals revealed strong positive spatial autocorrelation (Moran’s *I* = 0.79, *p* < 0.001), based on an 8-nearest-neighbour spatial weights matrix. This result indicates that, while MGWR captures part of the spatial structure in waterlogging occurrence, substantial spatially structured residual variation remains. This remaining spatial dependence motivated the development of the hybrid models to further improve predictive performance and reduce unexplained spatial clustering.

### Model comparison

Based on the spatial five-fold cross-validation, the hybrid frameworks exhibited distinct, metric-dependent trade-offs relative to the baseline MGWR (Table 3). In terms of discrimination, MGWR achieved an AUC of 0.672 ± 0.142, while XGBoost_MGWR yielded the highest AUC (0.755 ± 0.054), indicating improved ranking ability across locations. By contrast, MGWR_XGBoost showed a slightly lower AUC (0.663 ± 0.057) than MGWR, suggesting no additional gain in overall discrimination.

**Table 3:** The performance of hybrid models.

Model	AUC	Brier	MAE	RMSE	PR_AUC	Threshold	Sensitivity	Specificity
MGWR	0.672 ± 0.142	0.302 ± 0.154	0.514 ± 0.151	0.532 ± 0.153	0.536	0.588	0.833	0.417
XGBoost_MGWR	0.755 ± 0.054	0.371 ± 0.055	0.454 ± 0.053	0.608 ± 0.046	0.539	0.054	0.797	0.338
MGWR_XGBoost	0.663 ± 0.057	0.289 ± 0.039	0.466 ± 0.027	0.536 ± 0.037	0.576	0.649	0.463	0.734

For probabilistic accuracy, the pattern differed. MGWR_XGBoost produced the lowest Brier score (0.289 ± 0.039) and a reduced MAE (0.466 ± 0.027) compared with MGWR (0.302 ± 0.154; 0.514 ± 0.151), while maintaining a similar RMSE (0.536 ± 0.037 *vs.* 0.532 ± 0.153). In contrast, although XGBoost_MGWR achieved the lowest MAE (0.454 ± 0.053), it incurred the largest Brier (0.371 ± 0.055) and RMSE (0.608 ± 0.046), implying poorer overall probability reliability (*i.e.,* less accurate risk scores under the balanced case–control design) despite stronger discrimination.

Precision–recall analysis based on out-of-fold probabilities further highlighted these differences: MGWR_XGBoost attained the highest area under the precision recall (PR-AUC) (0.576), outperforming both MGWR (0.536) and XGBoost_MGWR (0.539). At the Youden-optimal thresholds (0.588 for MGWR, 0.054 for XGBoost_MGWR, and 0.649 for MGWR_XGBoost), MGWR delivered the highest sensitivity (0.833), XGBoost_MGWR remained relatively sensitivity-oriented (0.797) but with lower specificity (0.338), whereas MGWR_XGBoost emphasized specificity (0.734) at the cost of substantially reduced sensitivity (0.463). Overall, XGBoost_MGWR primarily improves discrimination, whereas MGWR_XGBoost improves probability accuracy and PR-oriented performance but shifts the operating point toward fewer false alarms and more missed events.

**Figure 7:**
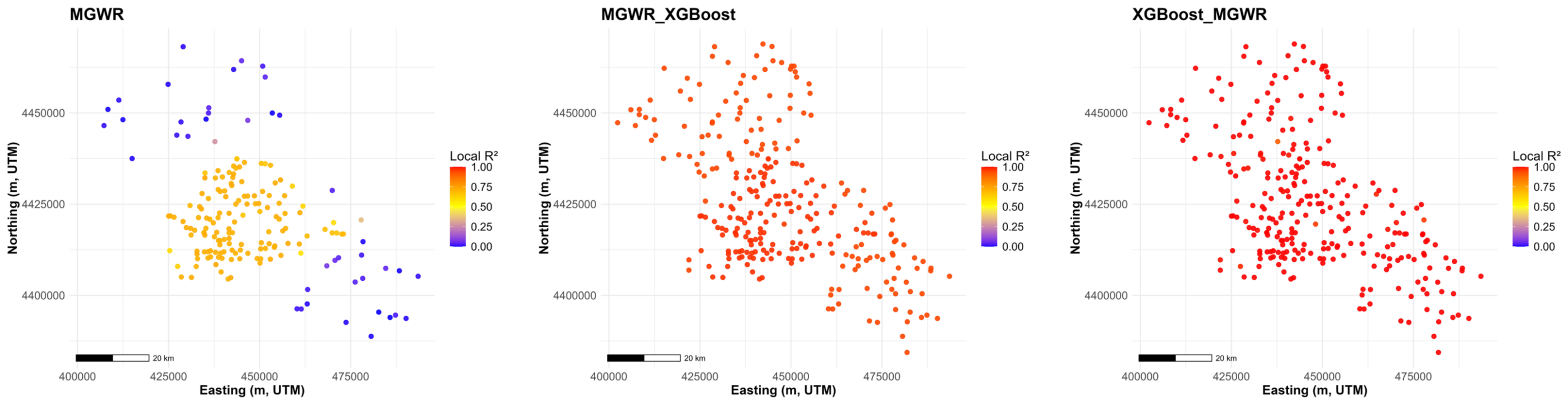
Spatial distribution of Local *R*^2^ values under different models (A) MGWR model, (B) MGWR_XGBoost model, and (C) XGBoost_MGWR model.

Spatial patterns of local *R*^2^ (Fig. 7) show that the baseline MGWR achieves relatively high explanatory power in the central part of the study area but leaves several peripheral clusters with low local *R*^2^. Both hybrid approaches substantially improve this spatial pattern by raising local *R*^2^ across most locations. The MGWR_XGBoost model yields consistently high local *R*^2^ over the domain with only minor spatial variation, while the XGBoost_MGWR model shows an even more uniformly high local *R*^2^ field, indicating that the hybrid corrections markedly enhance the spatial consistency of model fit relative to MGWR.

### Waterlogging risk prediction

**Figure 8:**
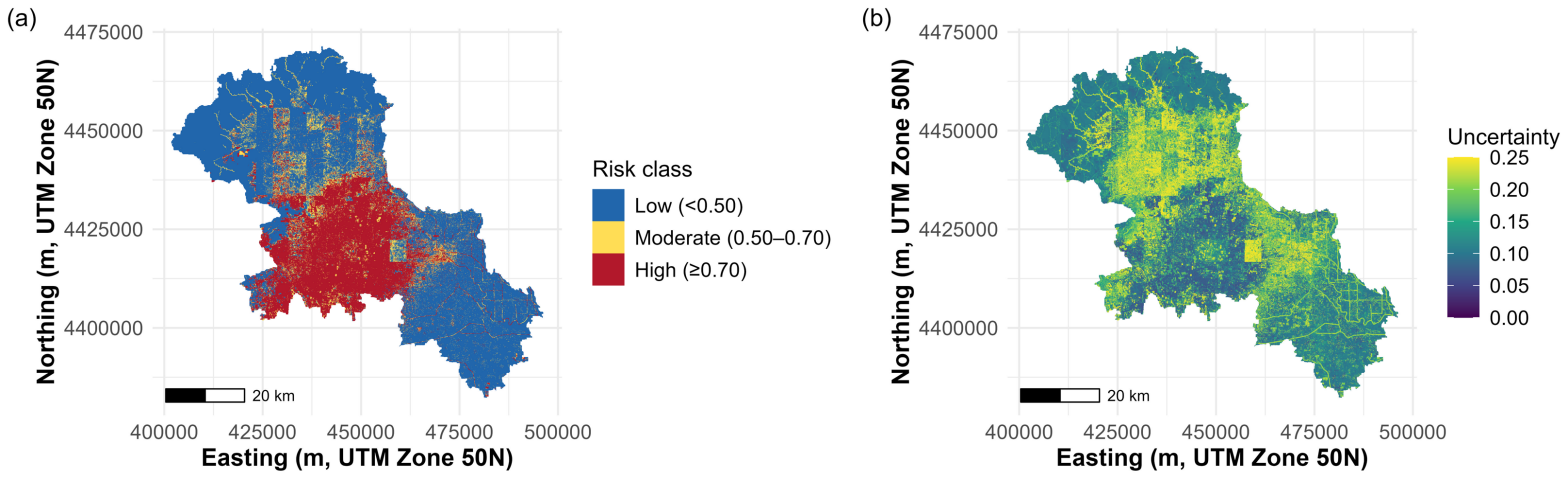
The map of waterlogging risk prediction. (A) Waterlogging risk probability; (B) waterlogging risk uncertainty.

The MGWR_XGBoost model delineates a clear and spatially coherent pattern of waterlogging risk (Fig. 8A). High-risk pixels (*p* ≥ 0.70) form a large contiguous core centered in the central–southern part of the study area. Surrounding this core, moderate-risk areas (0.50–0.70) appear primarily as a patchy transition zone—including dispersed bands and corridors around the core rather than a uniform ring—while the northern sector and the southeastern lobe are dominated by low-risk pixels (*p* < 0.50).

The corresponding uncertainty surface (Fig. 8B) is spatially heterogeneous, with uncertainty hotspots concentrated mainly in the northern portion of the study area and along linear corridor-like features (producing streaks of elevated uncertainty). In contrast, the central–southern high-risk core generally exhibits lower uncertainty, indicating more stable predictions in the region where risk is highest. Several peripheral areas show moderate uncertainty, suggesting that interpretation and prioritization should consider both predicted risk and uncertainty—particularly in northern and transitional zones where uncertainty is elevated despite moderate predicted risk.

## Discussion

### Hybrid model performance

Overall, the hybrid strategy provides a clear advantage over the standalone MGWR under spatially blocked evaluation. While MGWR captures spatially varying linear effects, adding a machine-learning component allows the framework to absorb residual nonlinearities and cross-factor interactions that MGWR alone cannot represent. Consequently, the two hybrid pipelines outperform MGWR in complementary ways—either by improving discrimination (XGBoost_MGWR) or by producing more reliable probabilities and better PR-oriented performance (MGWR_XGBoost) (Table 3). This pattern is consistent with the growing evidence that coupling machine-learning learners with geographically weighted models can strengthen urban waterlogging and pluvial-flood prediction (*e.g.*, Wang et al., 2023; Zhou et al., 2024; Yuan et al., 2024).

XGBoost_MGWR delivers the strongest discrimination (AUC = 0.755 ± 0.054) but the weakest probability reliability, with the highest Brier score (0.371 ± 0.055), the largest RMSE (0.608 ± 0.046), and low specificity (0.338). Its extremely low Youden-optimal threshold (0.054) further indicates a tendency to assign elevated probabilities broadly, which is undesirable for probability-based risk mapping. In contrast, MGWR_XGBoost provides a more mapping-oriented balance, achieving the lowest Brier score (0.289 ± 0.039), the highest PR-AUC (0.576), and markedly higher specificity (0.734) while maintaining competitive overall performance. Accordingly, because our primary goal is spatially consistent mapping with reliable probabilities and interpretable local effects, MGWR_XGBoost is adopted as the primary model for subsequent analyses.

Local *R*^2^ maps further diagnose spatial stability in model fit. The baseline MGWR exhibits moderate local *R*^2^ concentrated in the central cluster of sites, with noticeably lower values toward the northern and far southeastern tails, indicating reduced explanatory power where samples are sparse and covariate conditions depart from the core area (Fig. 7). Both hybrid pipelines substantially increase local *R*^2^ across the observations. Notably, XGBoost_MGWR produces an almost saturated field of near-unity local *R*^2^ with limited spatial contrast, whereas MGWR_XGBoost maintains consistently high local *R*^2^ while preserving smoother, more gradual spatial variation. This implies that local *R*^2^ can be overly optimistic for hybrid learners and is best interpreted alongside spatially robust cross-validation and probability-based metrics (Oshan et al., 2018; Chien, Carver & Comber, 2020).

### Effects of environmental drivers on waterlogging

The SHAP analysis of the XGBoost component reinforces that urban waterlogging in the study area is governed by a combination of topographic, land-cover and infrastructure-related factors rather than a single dominant control. At the global level (Fig. 3), ELE is by far the most influential predictor, followed by NPS and PRE, with FVC and River_dist, Road_dist playing secondary but still meaningful roles. This hierarchy is consistent with previous studies showing that flood and waterlogging hazards emerge from nonlinear interactions between terrain, built-up intensity and storm characteristics (Tong et al., 2023; Xiao et al., 2023), and confirms that machine-learning models can effectively capture these complex joint effects.

At the local level (Figs. 4 and 5), MGWR provides an explicit view of where covariate effects are spatially stable *versus* spatially heterogeneous. Among the eight predictors, elevation (ELE) clearly exhibits the strongest non-stationarity, with local coefficients spanning a much wider range than the other variables. High-magnitude ELE coefficients cluster along the central-to-southeastern corridor, while many locations elsewhere show coefficients close to zero, together indicating that micro-topography differentiates waterlogging risk primarily in specific subregions rather than uniformly across the entire study area.

In contrast, most other predictors display weak and spatially smooth coefficient surfaces. FVC, NPS, Slope, River_dist, and Road_dist are characterized by coefficients that remain close to zero across most observation sites, implying limited spatial differentiation in their marginal linear effects within MGWR. This does not contradict their global predictive contributions: SHAP-based importance reflects contribution to model prediction through potentially nonlinear and interaction effects, whereas MGWR coefficients represent local linear associations; therefore, their magnitudes and signs need not align one-to-one.

The generally muted local coefficients for vegetation and built-up proxies suggest that, within an already highly urbanized setting, these factors may act more as broad background conditions than as sharply varying local controls, and their flood-mitigation relevance likely depends on how green and grey infrastructure are integrated rather than on coverage alone (Green et al., 2021; Hou et al., 2022; Liu et al., 2025).

PRE and TR exhibit modest patchiness with localized positive and negative pockets, but their coefficient magnitudes remain small relative to ELE. This suggests that rainfall forcing and terrain roughness can modulate waterlogging probability in place-specific ways—potentially reflecting local storm concentration, surface routing, and drainage connectivity—yet they do not emerge as dominant spatially varying linear drivers in the MGWR component. Overall, the coefficient fields indicate that the spatially explicit signal captured by MGWR is mainly carried by micro-topographic variation, whereas most other predictors contribute more weakly or more uniformly in the local linear layer (Yang, Smith & Niyogi, 2019; Talwar & Yuan, 2024).

These patterns are consistent with the region’s broad terrain transition from the northwestern fringe toward the central and southeastern plain. The MGWR component attains higher explanatory power in the core built-up corridor (Fig. 7), where waterlogging observations are denser and where micro-topographic variability is more likely to interact with drainage capacity constraints. Peripheral zones—especially toward the margins—show lower local *R*^2^ under MGWR, indicating that additional nonlinearities and cross-factor interactions are more influential there and are better captured by the hybrid frameworks. Taken together, the results imply that the most consequential waterlogging hotspots are not determined by any single factor in isolation; rather, they emerge where micro-topography, storm exposure, and local infrastructure conditions co-occur, reinforcing pluvial flood susceptibility.

### Machine learning selection

Compared with RF, KNN and SVM, XGBoost achieved the best overall performance, with the highest AUC, accuracy and F1-score, while maintaining a balanced trade-off between precision and recall. RF ranked second, with slightly lower AUC and F1 but marginally higher recall at the expense of precision, indicating a greater tendency toward false positives and overestimation of waterlogging risk. KNN and SVM showed clearly lower AUC and accuracy; in particular, KNN’s relatively high recall but low precision implies many false alarms. These results indicate that XGBoost offers the most favorable balance between correctly identifying flooded locations and avoiding excessive false alarms, and we therefore selected it as the baseline classifier for subsequent hybrid modelling.

XGBoost’s superior performance can be attributed to its gradient-boosting framework, which iteratively corrects errors from previous models, enhancing predictive accuracy. Unlike RF, which relies on independent decision trees, XGBoost optimizes tree ensembles using gradient descent, improving efficiency and reducing bias. Additionally, XGBoost incorporates regularization (L1/L2) to prevent overfitting—a key advantage over KNN and RF, which are more prone to noise in high-dimensional data. Its built-in feature importance ranking also helps prioritize critical environmental variables, such as rainfall intensity and terrain elevation, leading to more interpretable flood-risk models. Previous studies have similarly demonstrated that XGBoost performs well in handling nonlinear relationships, further validating its effectiveness in this domain (Ren et al., 2023; Peng et al., 2025). Despite its strengths, XGBoost has limitations in flood-risk applications. Unlike geostatistical models (*e.g.*, kriging), it does not inherently account for spatial autocorrelation, requiring explicit spatial features for optimal performance.

### Evaluation validity and spatial leakage

In an initial screening of baseline machine-learning classifiers (RF, SVM, KNN, and XGBoost), cross-validation folds were generated by random partitioning without enforcing spatial separation between training and test samples. Because road-waterlogging events are spatially clustered within the eight core districts, such random resampling can yield optimistically biased estimates of predictive performance, particularly for flexible learners such as RF and XGBoost. Therefore, these random 10-fold CV metrics are interpreted only as non-spatial upper-bound performance for baseline model selection, rather than as spatially independent generalization accuracy (Xu et al., 2021).

To explicitly account for spatial dependence and avoid spatial leakage, all reported comparisons among MGWR and the two hybrid pipelines (XGBoost_MGWR and MGWR_XGBoost) were subsequently based on a spatially blocked k-fold evaluation, in which folds were defined by k-means clustering in geographical space and predictions were generated out-of-fold.

### Implications for urban waterlogging management

The proposed MGWR_XGBoost hybrid offers practical benefits for urban waterlogging governance in Beijing’s core districts. By combining a flexible machine-learning classifier with a spatially explicit regression component, it helps reconcile the usual trade-off between predictive accuracy and geographic interpretability. The resulting high-resolution maps of waterlogging probability and associated uncertainty provide an evidence base for proactive flood management, enabling planners and emergency services to identify robust hotspots of risk and to distinguish them from areas where model uncertainty remains high.

In operational terms, grid cells with predicted probabilities of road waterlogging ≥ 0.70 can be treated as structural priority zones for drainage upgrades, sewer rehabilitation and sponge-city interventions. Moderately exposed areas (0.50–0.70) with elevated uncertainty are better suited to intensified monitoring, targeted inspections and contingency planning, as both the potential for waterlogging and the model uncertainty are greatest there. The MGWR results further help distinguish locations where structural upgrades are likely to be most effective from those where improved maintenance and monitoring are more appropriate, supporting more targeted use of limited resources.

Beyond short-term mitigation, the hybrid framework also informs long-term resilience planning. By mapping the spatial variability of flood-driving factors within the eight-district study area, the results support climate-adaptive infrastructure design, including the strategic placement of green infrastructure and permeable surfaces where they are most likely to reduce surface ponding. At the same time, areas with systematically low local *R*^2^ and high residual clustering highlight locations where additional data on drainage capacity, sewer condition or maintenance practices are needed before firm design decisions can be made. Although calibrated here for Beijing’s urban core, the MGWR_XGBoost workflow is conceptually transferable to other cities, provided sufficient event records and basic environmental covariates are available, and thus offers a flexible template for data-informed pluvial flood risk management.

Taken together, our findings demonstrate that hybrid modelling approaches such as the MGWR_XGBoost framework can provide powerful tools for localized flood-risk mapping even when only a limited number of observed waterlogging events are available. By combining a flexible global learner (XGBoost) with a spatially structured MGWR adjustment, the framework effectively borrows strength from neighboring locations and continuous environmental fields, thereby enhancing predictive performance and stabilizing risk estimates under data-scarce conditions. In this sense, the proposed model contributes not only to high-resolution pluvial flood risk assessment in Beijing’s core districts, but also offers a transferable template for other cities where flood records are sparse but fine-scale risk information is urgently needed.

### Limitation and future

Several limitations should be acknowledged, offering opportunities for future research. First, the modelling design is based on an artificially balanced case–control sample, where non-flooded locations were generated to match the number of observed waterlogging points. This strategy is appropriate for comparing classifiers but implies that the predicted probabilities are not calibrated to the true prevalence of road waterlogging in the eight core districts. In this study, we therefore interpret the outputs primarily as relative risk scores rather than as exact occurrence probabilities. A natural extension would be to recalibrate the intercept or apply prevalence-based reweighting once more reliable city-wide estimates of the frequency of road waterlogging events become available.

Second, the model relies primarily on historical road waterlogging records, which may suffer from spatial clustering, incomplete coverage and positional uncertainty. In many cases, events are reported at intersections or approximate road segments, leading to location errors on the order of tens of meters. Such errors inevitably blur the relationship between waterlogging occurrence and fine-scale terrain or drainage features. Expanding and cross-validating event inventories through additional data sources—such as crowdsourced reports, social media feeds, municipal maintenance logs or high-resolution remote sensing imagery—could improve both the completeness and the spatial accuracy of flood records (Shoyama et al., 2020; Sadiq et al., 2022; Songchon, Wright & Beevers, 2023).

Third, while the covariates used here cover topography, land cover, and proximity measures, key drainage-capacity information (*e.g.*, sewer diameter/age/layout, inlet/manhole density and condition, detention storage) was unavailable at the required resolution. We therefore relied on river/road distance as coarse infrastructure proxies, which cannot fully represent hydraulic capacity or maintenance status. We also did not include digital elevation model (DEM)-derived hydrologic indices (*e.g.*, Topographic Wetness Index (TWI), flow accumulation) or drainage-density metrics because robust estimation in dense urban areas typically requires hydrologically conditioned high-resolution DEMs and detailed drainage-network data. Future work will integrate these drainage and flow-convergence indicators to better capture urban runoff routing beyond simple proximity measures.

Fourth, the present framework focuses on environmental predictors extracted around historical events. Yet urban pluvial flooding is inherently dynamic, governed by storm intensity, rainfall duration and the time-varying response of drainage systems (Bisht et al., 2016; Wang et al., 2022; Bibi, Reddythta & Kebebew, 2023). Coupling the MGWR_XGBoost framework with event-based hydrodynamic simulations or near-real-time rainfall and runoff information would be a promising step towards operational early warning and nowcasting.

Fifth, we did not systematically explore broader socio-economic and planning determinants—such as zoning regulations, population density, land-development history or infrastructure investment patterns—that may shape exposure and vulnerability (Salazar-Briones et al., 2020; Hemmati et al., 2021; Islam & Meng, 2024; Li et al., 2025). Extending the framework to include these variables would enable a more holistic understanding of how physical hazards, infrastructure and social context jointly produce urban waterlogging risk. Finally, although the model currently provides robust risk predictions and interpretable driver patterns, it has not yet been applied to scenario analysis or optimization of infrastructure interventions. Future studies could build on this framework to evaluate alternative drainage upgrade plans or sponge-city layouts, thereby offering more direct decision support for integrated urban waterlogging mitigation.

Sixth, the predictors used in this study have heterogeneous native spatial resolutions. Although coarse products were resampled to a 30 m grid for stacking and wall-to-wall mapping, this step is primarily a computational alignment and does not introduce new fine-scale information. Consequently, coarse-resolution predictors mainly contribute broad-scale background gradients, which may affect global importance diagnostics (*e.g.*, SHAP-based rankings) and constrain the effective spatial detail that can be attributed to the 30 m probability map. In future work, incorporating higher-resolution hydrometeorological and land-surface products (*e.g.*, finer soil moisture, rainfall, and drainage-related proxies) would help reduce this scale mismatch, improve the fidelity of local driver interpretation, and better support truly fine-grained risk delineation.

## Conclusion

Using spatially blocked five-fold cross-validation to avoid spatial leakage, we show that hybridizing MGWR with machine learning improves model utility for urban waterlogging mapping, albeit with clear, metric-dependent trade-offs. XGBoost_MGWR provides the strongest discrimination (AUC = 0.755 ± 0.054) yet shows weaker probability reliability (Brier = 0.371 ± 0.055; RMSE = 0.608 ± 0.046) and low specificity, with an extremely low Youden-optimal threshold indicating a tendency to assign elevated probabilities broadly. In contrast, MGWR_XGBoost offers a more mapping-oriented balance, achieving the best probability accuracy and PR performance (Brier = 0.289 ± 0.039; PR-AUC = 0.576) and substantially higher specificity (0.734), while retaining stable spatial fit patterns. Accordingly, we selected MGWR_XGBoost to generate the final risk map, as it provides more reliable risk scores and higher probabilistic accuracy while maintaining a spatially consistent fit, which is more suitable for mapping applications that require interpretable risk levels.

Urban waterlogging in Beijing reflects the combined influence of multiple environmental drivers rather than a single dominant control. The proposed framework translates these relationships into high-resolution maps of risk and uncertainty, distinguishing high-probability hotspots from moderate-probability areas where prediction uncertainty is higher. These moderate-risk, higher-uncertainty areas are practical priorities for field verification, drainage diagnostics, and incremental infrastructure upgrading.

Taken together, the MGWR_XGBoost approach demonstrates how combining spatially explicit regression with flexible machine learning can enhance localized pluvial flood risk mapping in complex, data-constrained urban settings. Beyond Beijing, the framework is readily transferable to other cities where flood observations are limited but spatially referenced environmental data are available. By improving the spatial reliability of risk estimates under sparse and clustered samples, the proposed method offers a practical, scalable tool to support targeted drainage investment, sponge-city design and evidence-based urban flood resilience planning under a changing climate.

## Supplemental Information

10.7717/peerj.20977/supp-1 Supplemental Information 1 Supplemental Tables

10.7717/peerj.20977/supp-2 Supplemental Information 2 The raw data including flooded and non-flooded points

## Additional Information and Declarations

### Data Availability

The following information was supplied regarding data availability: The raw data is available in the Supplemental File.
