# Supplementary material for: A spatially interpretable machine learning framework for urban waterlogging risk mapping in Beijing
Source: PeerJ. 2026 Mar 19;14:e20977. doi: 10.7717/peerj.20977 (PMC13006009; doi:10.7717/peerj.20977)
Supplement: Supplemental Information 1 [file peerj-14-20977-s001.docx]

Table S1. Sensitivity of model performance to the minimum distance threshold used for negative sample generation.

| Distance | AUC | RMSE | MAE | Brier |
| --- | --- | --- | --- | --- |
| 1km | 0.825±0.061 | 0.413±0.035 | 0.352±0.032 | 0.171±0.028 |
| 2km | 0.857±0.057 | 0.392±0.054 | 0.301±0.047 | 0.156±0.041 |
| 3km | 0.909±0.070 | 0.347±0.071 | 0.262±0.053 | 0.125±0.048 |

**Notes:** This sensitivity analysis evaluates the robustness of model performance to the choice of minimum separation distance between flooded and non-flooded samples. Negative samples were generated using minimum distances of 1 km, 2 km, and 3 km from any flooded point. Performance metrics are reported as mean ± standard deviation across cross-validation folds. Although larger distances tend to yield higher apparent performance, all results were derived using the same modelling workflow, indicating that the relative ranking of models is robust to the distance threshold.

Table S2. Comparison of MGWR kernel functions and optimal bandwidth selection.

| Kernel | Optimal_BW | AIC | AICc | BIC | RSS | R2 | R2adj |
| --- | --- | --- | --- | --- | --- | --- | --- |
| bisquare | 67.000 | -488.937 | -181.543 | -215.469 | 1.806 | 0.974 | 0.944 |
| gaussian | 20.000 | -32.223 | 28.321 | -114.242 | 12.399 | 0.821 | 0.775 |
| exponential | 20.000 | 2.886 | 80.257 | -38.822 | 13.638 | 0.803 | 0.727 |
| tricube | 64.000 | -392.633 | -190.081 | -227.769 | 2.784 | 0.960 | 0.932 |

Notes: This table reports diagnostic statistics for MGWR models fitted using alternative kernel functions. The bisquare kernel achieved the best overall fit and was therefore adopted in the main analysis.
